# Drought Yield QTL (*qDTY*) with Consistent Effects on Morphological and Agronomical Traits of Two Populations of New Rice (*Oryza sativa*) Lines

**DOI:** 10.3390/plants8060186

**Published:** 2019-06-24

**Authors:** Asmuni Mohd Ikmal, Zainuddin Nurasyikin, Tuan Ali Tuan Nur Aqlili Riana, Zulkafli Puteri Dinie Ellina, Ratnam Wickneswari, Abd Aziz Shamsudin Noraziyah

**Affiliations:** Faculty of Science and Technology, Universiti Kebangsaan Malaysia, Bangi 43600, Malaysia; mohdikmal@siswa.ukm.edu.my (A.M.I.); asyikinzainuddin@gmail.com (Z.N.); aqlilyryana1912@gmail.com (T.A.T.N.A.R.); p93778@siswa.ukm.edu.my (Z.P.D.E.); wicki@ukm.edu.my (R.W.)

**Keywords:** agronomy, drought, morphology, pyramided lines, QTL

## Abstract

Drought has been a major limiting factor for rice production. Drought yield QTLs (*qDTYs; QTLs =* quantitative trait loci) were pyramided into MRQ74 and MR219 to produce drought tolerant lines. In this study, new drought tolerant MRQ74 and MR219 pyramided lines (PLs) were evaluated under drought stress (RS) and non-stress (NS) conditions to evaluate the effects of different *qDTYs* combinations on morphological and agronomical traits. MRQ74 PLs having *qDTY_12.1_* possessed the best root length (RL) under both RS and NS but the effect was only significant for MR219 PLs under RS. Some *qDTYs* combinations also found to have consistent effect on the same trait of both populations. PLs with only *qDTY_12.1_* showed the highest grain yield (GY) under RS in both populations which means *qDTY_12.1_* controlled RL and caused higher GY under drought condition. The interaction of major-effect *qDTY_12.1_* with *qDTY_2.2_* also shows significant effect on leaf rolling (LR) of both PL populations. These *qDTYs* proved to be beneficial in improving traits related to drought tolerance. Selected PLs with *qDTY_12.1_* combinations also found to have better RL and root weight (RW) under RS. Improvement of morphological and agronomical traits led to higher GY of PLs. Therefore, *qDTY_12.1_* either is present singly or in combination with other *qDTYs* was the best *qDTY* due to its consistent effect on morphological and agronomical traits and GY across populations under RS and NS.

## 1. Introduction

Rice, which was produced 600 million tons on an area of more than 150 million hectares (ha), has become the main source of daily calories for more than 3.5 billion people in Asia, Africa and Latin America [1,2]. In Malaysia, each Malaysian consumed 78.8 kg rice per year on average. For the past few years, Malaysia’s rice self-sufficiency level (SSL) was at 71% and average yield was around 3.0 to 3.5 tonnes per hectare. To add the shortage of 29% SSL, rice were imported from countries such as Thailand, Vietnam, Pakistan and India. Malaysia population size is increasing year by year and the latest figure in 2016 was 31.7 million. Production of rice in local rice fields needs to be increased to feed the population. However, the aim to achieve 100% SSL by 2020 has high probability to fail due to more adverse climate conditions happening recently. Drought, which is perilous to rice can thwart the effort to increase rice production and would drive bad consequences to national food security.

Drought will affect all rice growth stages but the effect is much more severe during early reproductive stages especially during anthesis [3,4]. While good irrigation system was equipped in the granary areas, most rice fields managed by individual farmers lack this feature and will cause devastative effects to their fields. Most paddy farmers in southern part of peninsular Malaysia experienced low amount of rain, which was 40–60% lower than the average amount in the month of February 2017, which is supposed to be the main planting season. Therefore, drought tolerant rice with desirable phenotypes and higher yield than current cultivars need to be developed in Malaysia to counteract the effect of water scarcity.

Numerous efforts to produce drought tolerant rice have been done around the world but in Malaysia, the effort seems to be slow and no drought tolerant rice declared until now. Influence of environment, polygenic property of drought related traits, and inadequate criteria to select drought tolerance traits further increase the complexity of producing drought tolerant rice [5,6]. While many reports suggested secondary trait as selection criteria for enhancing drought tolerance, grain yield is still the most suitable trait to improve drought yield due to its moderate to high heritability under drought [2]. The evidence from several studies has confirmed that direct selection for GY under drought is an effective criterion in drought breeding program [7,8,9,10]. Detection of major effect drought yield quantitative trait loci (QTLs) related to yield under drought (*qDTYs*) by researchers around the world, mainly in International Rice Research Institute (IRRI), has provided better approach to breed rice adaptable to drought. 

A large and consistent effect *qDTY_12.1_* was identified by Bernier et al. [11] in a population of Vandana/Way Rarem, Ghimire et al. [12] and Vikram et al. [13] detected major effect *qDTY_1.1_* in several populations, *qDTY_2.2_*, *qDTY_4.1_*, *qDTY_9.1_* and *qDTY_10.1_* were detected in population of AdaySel/IR64 [14]. All these *qDTYs* were introgressed or pyramided into mega-varieties through marker assisted breeding (MAB) to enhance tolerance to drought. However, until now, only two of Malaysia’s cultivars which are MR219 and MRQ74 reported to be pyramided with *qDTYs* (*qDTY_2.2_*, *qDTY_3.1_*, *qDTY_12.1_*) [15,16]. Those *qDTYs* were reported to give pyramided lines (PLs) yield advantage of 903 to 2500 kg ha^−1^ over recipient parent MR219 [15] and 1009 to 3473 kg ha^−1^ over recipient parent MRQ74 [16] under drought. This study focused on evaluating the drought tolerant pyramided lines (PLs) of MRQ74 and MR219 under RS and NS with the objectives to identify morphological and agronomical traits which respond to RS, to identify morphological and agronomical traits which are related to different combinations of *qDTYs* and to identify *qDTY*/s with the most effective and consistent effects on both PL populations under NS and RS.

## 2. Results

### 2.1. Drought Imposition

**Figure 1:**
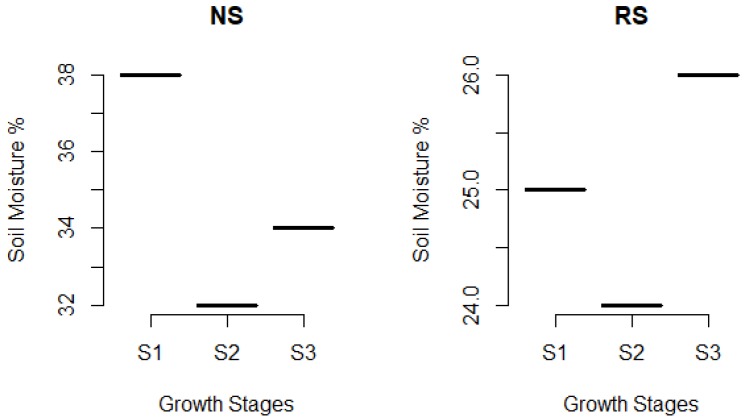
Soil moisture content in non-stress (NS) and drought stress (RS) plot. S1: Early reproductive, S2: Reproductive, S3: Maturity.

During period of drought imposition, several days have recorded high amount of rain but no stagnant water was allowed in the plot. Water level in RS plot ranged from −15 to −51 cm. Soil moisture measurement using gravimetric method always recorded lower soil moisture in RS plot compared to NS plot (Figure 1).

### 2.2. Assortment of PLs into qDTY Classes

According to scoring data obtained after polyacrylamide gel electrophoresis (PAGE), PLs were assorted into their respective *qDTY* classes. For MRQ74 PLs, eleven were in class A (*qDTY_12.1_* + *qDTY_3.1_* + *qDTY_2.2_*), two in classes B (*qDTY_12.1_* + *qDTY_3.1_*) and G (*qDTY_12.1_* + *qDTY_3.1_* + *qDTY_2.2_*), four in classes C (*qDTY_12.1_* + *qDTY_2.2_*), E (*qDTY_12.1_*) and F (*qDTY_3.1_*), and eight in class D (*qDTY_3.1_* + *qDTY_2.2_*). While for MR219 PLs, six in class A, ten in class B and D, three in class C, one in class E and four in class F. 

### 2.3. Effects of qDTY Classes

Analysis of *qDTY* classes showed that no similar trait in both populations controlled by one *qDTY* class under NS. Three traits were found to be controlled by one *qDTY* class under RS namely LR, RW and GY. LR under RS was controlled by class B, which had the lowest LR score compared to other *qDTYs* and to MRQ74 (Table 1 and Table 2). RW were highly affected by the presence of *qDTY* class E. MRQ74 PLs with class E had mean RW of 2.31 g while MR219 PLs had mean RW of 0.90 g. RW of this *qDTY* class is lower than MRQ74 (3.66 g) but among the *qDTY* classes, it was still the best. Class E also played role in controlling GY of PLs under RS. PLs with class E recorded highest mean of GY compared to the other *qDTY* classes, which were 1826.58 kg ha^−1^ in MRQ74 population and 2788.62 kg ha^−1^ in MR219 population. This *qDTY* class had respective yield advantage (YA) of 208.52 kg ha^−1^ and 782.91 kg ha^−1^ over MRQ74 and MR219.

While those traits were shown to be affected by the same *qDTY* classes in both populations, the other traits were controlled by different *qDTY* classes. Under NS, RW of MR219 PLs and MRQ74 PLs were controlled by class C and class E respectively. RL and GY of MR219 PLs were best affected by class A but these traits in MRQ74 PLs were governed respectively by different *qDTY* classes which were class E and D. Class A of MR219 PLs recorded YA of 416.08 kg ha^−1^ over MR219 but MRQ74 have higher GY compared to PLs in all *qDTY* classes which might be due to some PLs that have very low yield. Number of panicles (NP) and plant height (PH) of these two populations also influenced by different *qDTY* classes where class E for MR219 PLs while class C and D respectively for MRQ74 PLs. The 100-grain weight (GW) of MR219 PLs were controlled by class D while class G controlled GW of MRQ74 PLs. The effect of class D can be seen to reduce DTF of the MR219 PLs compared to MR219. 

Under RS, the effect of *qDTY* classes to PH and NP of these populations were likely to interchange. NP and PH of MR219 PLs were influenced by class C and E respectively whereby in MRQ74 PLs, the other way around happened. RL of both PLs population also affected by different *qDTY* classes where class E showed the best effect to MR219 PLs while class G to MRQ74 PLs. As happened in NS, GW under RS also governed by different *qDTY* classes.

Class C showed the best effect to MRQ74 PLs while class F to MR219 PLs. Days to 50% flowering (DTF) and chlorophyll content (CC) of MR219 PLs were highest in class D while these traits were controlled by class F in MRQ74 PLs. MR219 PLs with *qDTY_3.1_* + *qDTY_2.2_* have improved DTF under RS than MR219. 

Some *qDTY* classes also showed consistent effect to similar traits in NS and RS. For instance, result of *qDTY* class analysis in Table 2 showed that PH of MR219 PLs was best affected by class E. Effects of class D to DTF and CC and class E to PH of MR219 PLs were also consistent in both NS and RS. Class F had shown consistent effect to CC of MRQ74 PLs in which it was the best *qDTY* class in both conditions. Effect of class E to RW also consistent in MRQ74 PLs under NS and RS. Grain length (GL) under RS for MRQ74 PLs and MR219 PLs population were greatly influenced by the *qDTY* class C. 

There was varying trend in NP of both populations under NS and RS but none of them showed drastic changes. There are significant differences observed for NP of MR219 PLs under RS compared to MR219 where MR219 had more NP. The same situation was also observed in MRQ74 PLs where NP of PLs were lower than MRQ74 but the difference was not significant. The result also showed that combination of the two *qDTYs*, *qDTY_3.1_* + *qDTY_2.2_* was effective in enhancing the NP under NS thus resulting in increased of GY. 

All *qDTY* classes showed longer RL in RS compared to NS. MRQ74 PLs having *qDTY_12.1_* possessed the best RL and RW under both conditions. Moreover, *qDTY_12.1_* also affecting RL and RW of MR219 PLs but the best effect can only be seen under RS. 

As shown in Table 1 the effect of *qDTY_2.2_* as single QTL to MRQ74 PLs under RS and NS was not so good compared to the other *qDTYs* classes. Combination *qDTY_2.2_* + *qDTY_3.1_* showed the greatest effect to GY under NS. In the MR219 PLs, no PLs were found to have *qDTY_2.2_* that exist as single *qDTY*. Combination of *qDTY_2.2_* with the other two *qDTYs* studied (*qDTY_12.2_* + *qDTY_3.1_* + *qDTY_2.2_*) found to be the best *qDTY* class under NS and the second best under RS. 

### 2.4. Performance of Selected MRQ74 PLs and MR219 PLs

**Figure 2:**
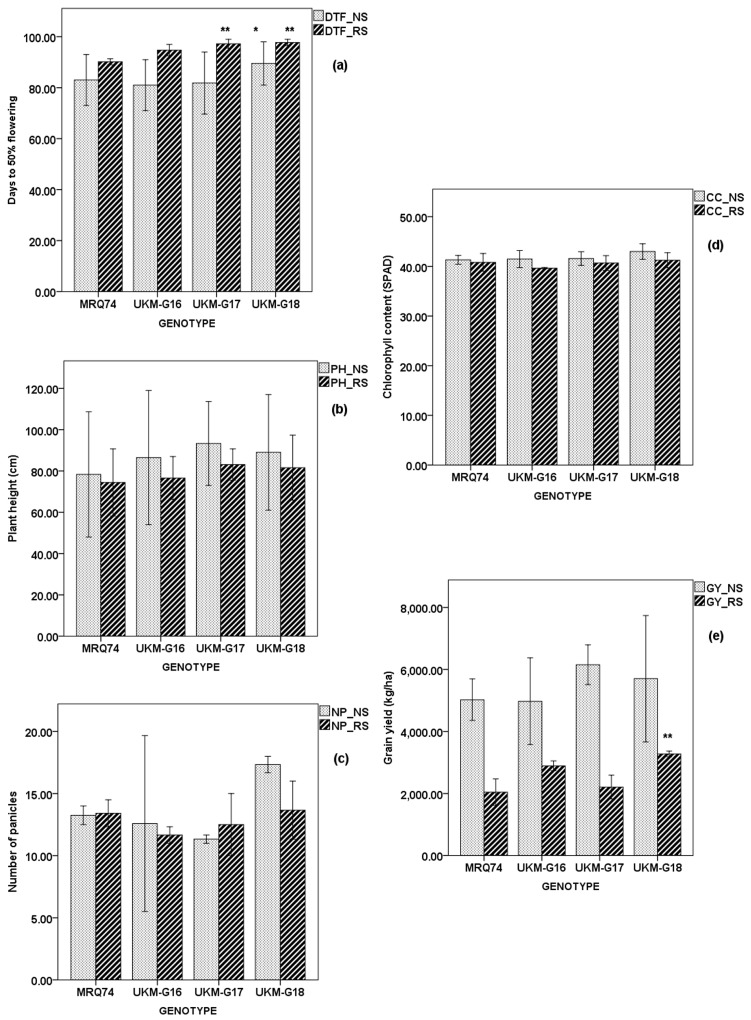
Average of mean of MRQ74 population for two seasons under NS and RS. (**a**) Days to 50% flowering, (**b**) Plant height (cm), (**c**) Number of panicles, (**d**) Chlorophyll content, (**e**) Grain yield (kg/ha). * Significant difference from MRQ74 under NS; *p* < 0.05, ** Significant difference from MRQ74 under RS; *p* < 0.05. Error bar: ± standard error (SE).

To confirm the effect of these *qDTYs*, few PLs for each population were selected based on their morphological traits in field such as size of tillers, height and lodging resistance. Selection were done by Department of Agriculture Malaysia without knowing the genetic content of the PLs. Averaged data of two planting seasons (Figure 2) showed that IR98010-126-18-4-1-1-1 (UKM-G17) with class C (*qDTY_12.1_* + *qDTY_2.2_*) was the highest yielding MRQ74 PL under NS (6278.91 kg ha^−1^), 1119.63 kg ha^−1^ yield advantage (YA) over MRQ74. IR98010-134-4-1-2-1-1 (UKM-G18) with class G (*qDTY_2.2_*) recorded highest GY (3261.53 kg ha^−1^) which was significantly different compared to MRQ74 under RS where the YA was 1131.28 kg ha^−1^ over MRQ74. UKM-G17 had the longest RL and RW under NS and RS compared to the other selected PLs (Supplementary Tables S1 and S2). Under NS, IR98008-103-77-1-4-1-1 (UKM-G16) recorded the earliest DTF, which was 79.60 days while UKM-G18 was the latest to flower (87.80 days).

**Figure 3:**
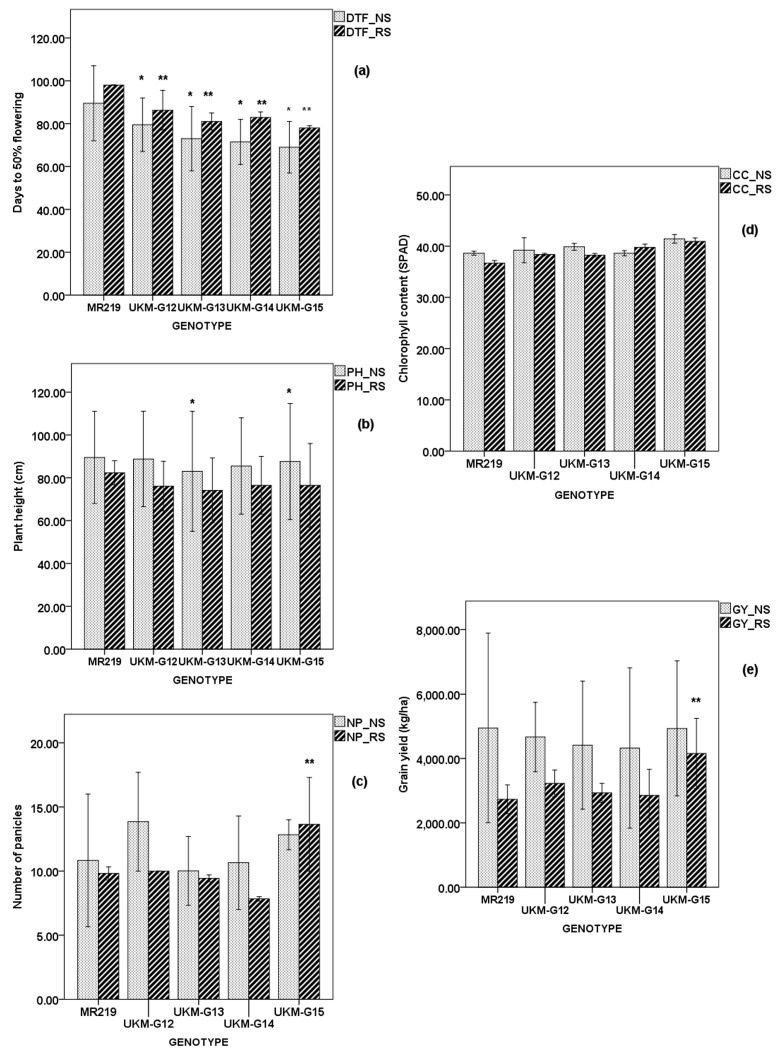
Average of mean of MR219 population for two seasons under NS and RS. (**a**) Days to 50% flowering, (**b**) Plant height (cm), (**c**) Number of panicles, (**d**) Chlorophyll content, (**e**) Grain yield (kg/ha). * Significant difference from MR219 under NS; *p* < 0.05, ** Significant difference from MR219 under RS; *p* < 0.05. Error bar: ± standard error (SE).

IR99784-226-335-1-5-1-1 (UKM-G12) and IR99784-255-91-1-1-1-1 (UKM-G15) which had *qDTY_12.1_* + *qDTY_3.1_* + *qDTY_2.2_* and *qDTY_3.1_* + *qDTY_2.2_* respectively produced higher GY than MR219 under NS with YA of 1041.80 kg ha^−1^ and 184.20 kg ha^−1^ (Figure 3). Under RS, UKM-G12 and UKM-G15 also produced higher GY than MR219 (Figure 3) with YA of 601.54 kg ha^−1^ and 1573.70 kg ha^−1^ respectively in which GY of UKM-G15 was significantly different from MR219. These two PLs have good performance under both conditions and will be further tested in several locations. IR99784-255-68-1-7-1-1 (UKM-G13) had the longest RL and heaviest RW among the selected PLs under RS but not under well irrigated condition (Supplementary Tables S3 and S4).

All PLs and MR219 had increased DTF under RS but greatest delay was observed on MR219, which delayed for 12 days. All PLs’ DTF were significantly different to MR219 under both conditions. No significant difference of PH was found in both selected PL populations under RS and MRQ74 PLs population under NS but not in MR219 PLs under NS. Two selected lines, which were UKM-G13 and UKM-G15 recorded significant PH differences compared to MR219. All genotypes have reduced PH under RS compared to NS in both populations. In MR219 population, greatest reduction was witnessed on PH of UKM-G12 where it reduced by 14.80 cm under RS compared to under NS. PH of UKM-G13, UKM-G14 and UKM-G15 under RS were reduced by 11.60 cm, 12.00 cm and 8.60 cm respectively. Greatest PH reduction on MRQ74 population was seen on UKM-G16 where PH reduced by 16.33 cm. PH of UKM-G17, UKM-G18 and MRQ74 reduced by 14.60, 9.87 and 6.73 cm respectively. 

MR219 PL with the highest NP under NS was UKM-G13 with 13.53 panicles while highest NP under RS was recorded by UKM-G15 with 14.40 panicles. MR219 PLs and MR219 have reduced NP under RS compared to NS except UKM-G15 and was significantly different from MR219. As seen in MR219 population, MRQ74 PLs and MRQ74 have reduced NP under RS compared to NS except UKM-G17. UKM-G16 and UKM-G17 had lower NP than MRQ74 under RS but the differences were not significantly different. 

Even though CC expected to reduce under RS, only UKM-G13, UKM-G14 and MR219 had lower CC under NS compared to RS. CC of the other two PLs, UKM-G12 and UKM-G15 were increased by 0.71 and 1.02 respectively. Meanwhile, all MRQ74 PLs and MRQ74 experienced reduction of CC under RS. Reductions were ranged from 0.77 to 1.74. All MRQ74 PLs have higher CC than MRQ74 except UKM-G16 but were not significantly different. 

### 2.5. Correlation between Traits under NS and RS

**Figure 4:**
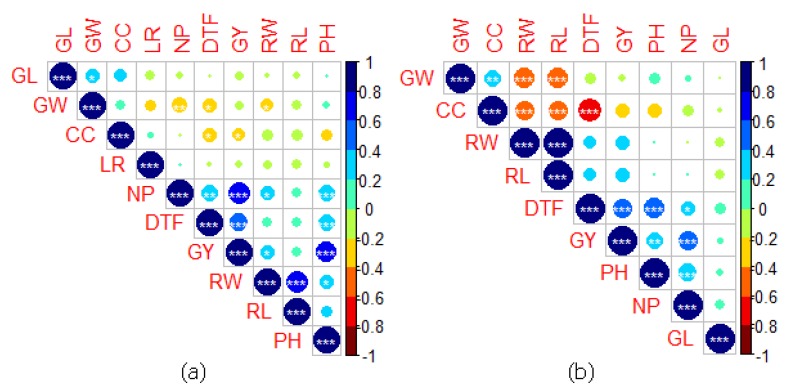
Correlation matrix of traits evaluated. (**a**) MRQ74 pyramided lines (PLs) under RS, (**b**) MRQ74 PLs under NS, Days to 50% flowering (DTF), plant height (PH) in cm, number of panicles (NP), chlorophyll content (CC), leaf rolling (LR), root weight (RW) in g, root length (RL) in cm, grain weight (GW) in g, grain length (GL) in cm, grain yield (GY) in kg ha^−1^. * *p* < 0.05, ** *p* < 0.01, ** *p* < 0.001.

**Figure 5:**
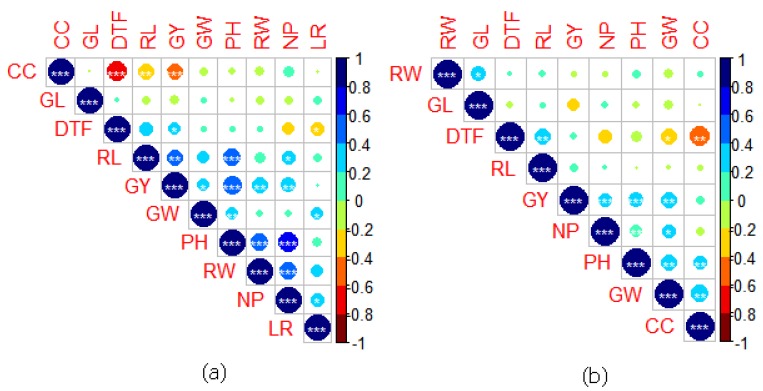
Correlation matrix of traits evaluated. (**a**) MR219 PLs under RS, (**b**) MR219 PLs under NS, Days to 50% flowering (DTF), plant height (PH) in cm, number of panicles (NP), chlorophyll content (CC), leaf rolling (LR), root weight (RW) in g, root length (RL) in cm, grain weight (GW) in g, grain length (GL) in cm, grain yield (GY) in kg ha^−1^. * *p* < 0.05, ** *p* < 0.01, ** *p* < 0.001.

Under RS, GY of MRQ74 PLs was positively correlated with DTF, PH, NP, RW and RL but negatively correlated with CC, LR, GW and GL (Figure 4). However, only DTF, PH, NP and RW showed significant correlation (*p* < 0.05). For MR219 PLs, GY was positively correlated with DTF, PH, NP, RW, RL, LR and GW (Figure 5). Only CC showed significant negative correlation with GY. As expected, positive correlation also recorded between RW and GY [*r* = 0.26 (MRQ74 PLs), *r* = 0.33 (MR219 PLs)] as well as between RL and GY [*r* = 0.15 (MRQ74 PLs), *r* = 0.37 (MR219 PLs)] under RS. Under NS, GY of MRQ74 PLs was positively correlated with DTF, PH, NP, RW, RL and GL. For MR219 PLs, GY recorded positive correlation with DTF, PH, NP, CC, RL and GW. Numerical values of correlation were provided in Supplementary Tables S5 and S6.

### 2.6. Heritability of Traits under NS and RS

Most traits of MRQ74 PLs have high heritability (*H*) except GL (*H* = 0.05) under NS. *H* for all traits decreased under RS except GW where *H* increased from 0.05 to 0.53. *H* of MR219 PLs under NS ranged from 0.00 to 0.99 in which the lowest was recorded by RW. Unlike MRQ74 PLs, *H* of MR219 PLs’ GY was slightly higher in RS than in NS (Table 3).

## 3. Discussion

Drought imposition in this study was considered mild or moderate since level of water depth did not reach −100 cm. However, stress imposed during the second planting season in Perak was classified as severe. We found that the effect of every *qDTY* classes to any traits of both PL populations seem to be different among population and between NS and RS. Based on overall results, PLs with *qDTY*/s performed better than the parents and contributed to better performance of *qDTY* classes under NS and RS. Some *qDTY* classes showed effect to certain trait on MR219 PLs but not to MRQ74 PLs and vice versa. This condition might be due to different expression of these *qDTYs* in different genetic background. Pleiotropic effects also can be suggested to occur due to several traits shown to be affected by a single *qDTY* such as *qDTY_12.1_* which affects PH, RW, RL and GY. Gene underlying this *qDTY* might be responsible for expression of these traits under RS. Traits that were associated with drought tolerance such as DTF, LR were improved with the presence of *qDTY*. For instance, combination of *qDTY_3.1_* with *qDTY_2.2_* was significant towards improvement DTF. Previous study also found that *qDTY_3.1_* had large effect on DTF under moderate stress [17]. 

Apart from that, all *qDTY* classes showed reduction of CC in RS compared to NS which indicated great effect of RS to CC [17,18]. Perhaps, RS interrupted pigment synthesis pathways and degradation, loss of chloroplast membrane and lipid peroxidation which further lead to CC reduction [19]. CC will be affected either directly or indirectly by moisture, nutrient and energy deficit which disrupt some physiological processes [20]. As can be seen in Table 1 and Table 2, although PH was expected to decrease under RS, only class F in MRQ74 PLs, class D and E in MR219 PLs showed reduction of PH while the others increased. Drought is considered mild in this study that the effect is not significant towards reduction of PH. Plants will stop or slow down growth during occurrence of stress and cause elongation and expansion of cells to be stunted. Due to reduction in tugor pressure, growth of cell is severely impaired under stress [19]. Classes with reduced PH under stress might have experienced severe stress due to the inability to maintain optimum level of tugor pressure to continue growth. Several previous studies also recorded reduction of PH under RS [21,22].

Plant rolling leaves to maintain water status [23], however rolled leaves indicate inadequacy of water in the cells which further lead to loss of tugor pressure [24]. Different genotypes will have different mechanism behind LR as Pandey & Shukla [19] mentioned that genetic plays role towards existence of variation of LR score between genotypes since previous studies had found specific QTL controlling LR. MRQ74 scored high LR in this study, which means it was experiencing greater water stress than PLs. LR give bad effect to plant as rolled leaves will reduce leaf surface area exposed to light for photosynthesis to occur which then lead to low grain filling. While it is suggested that lower LR score will contribute to higher GY, results obtained in this study did not show any significant relationship between those two traits, which showed the effect of LR is very low. Meanwhile, Kadioglu & Terzi [25] described leaf rolling as a mechanism for plant to prevent dehydration and reduce transpiration rate but instant rolling during water stress is an indicator of low ability to maintain cell tugor pressure. MR219 has the lowest LR score compared to the *qDTY* classes which suggest that MR219 did not utilize the leaf rolling mechanism to conserve water. The effect of dehydration might cannot be seen on the leaves of MR219 but might affect other parts such as the flower which further lead to low grain filling. 

Since PLs with *qDTY_12.1_* had better RL and RW, *qDTY_12.1_* can be inferred as the most beneficial *qDTY* in affecting root traits when it presents alone without combination of other *qDTYs*. Dixit et al. [26] also reported increase of transpiration efficiency, panicle and root branching and GY of near isogenic lines (NILs) with *qDTY_12.1_* under RS. Gowda et al. [27] highlighted that high root penetration and branching ability as vital traits for rice to avoid drought. Reduction of photosynthesis rate also witnessed before due to reduction of RW and RL [28]. Root traits is vital for developmental processes especially during water scarcity. Ability of plant to acquire water under drought stress contributes to better physiological processes, growth and development. Drought avoidance, which is one of drought mechanism also contributed by root structure such as deep and coarse root. High RW and RL of PLs may be due to increased branching and diameter which lead to better penetration ability [29,30]. Concentration of Abscisic acid (ABA) which known to be the phytohormone that related to drought stress in plant increases during water stress [20] and due to this condition, root growth will be induced for plant to gain more water. This might be one of the reasons, which lead to better root traits under RS. As LR was affected by *qDTY_12.1_* + *qDTY_3.1_*, the presence of *qDTY_12.1_* that controlled RL and RW might be the reason for better LR under RS. High RL and RW facilitate plants to obtain more water from the soil thus increasing leaf water status. 

In this study, *qDTY_2.2_* was contributed by NIL developed from a cross between Aday Sel and IR64. Previous studies had found this *qDTY* to show large effect in the background of IR64 under RS with varying severities [14]. In contrast, a study conducted by Palanog et al. [31] found that *qDTY_2.2_* which contributed by Kali Aus, a drought tolerant traditional variety from India did not showed effect to lines with IR64 background but the effects were seen against MTU1010 background. Effect of *qDTY_2.2_* singly in the background of MR219 and MRQ74 was not so good on GY under RS and NS. This is not surprising as Palanog et al. [31] had mentioned before that the expression of QTL is dependent on genetic background and source of QTL.

Based on overall recorded and observed traits, *qDTY_12.1_* was chosen as the best *qDTY* for both MRQ74 PLs and MR219 PLs population. This *qDTY_12.1_* showed consistent effect on traits under RS and NS. Presence of *qDTY_12.1_* improved RL and RW where these traits often associated with the ability of plants to obtain water under drought. Growth of lateral roots which contributed by *qDTY_12.1_* further explain the effect of this *qDTY* to better GY under RS [32] (Henry et al. 2015). Researches by Bernier et al. [7] and Mishra et al. [33] also found that *qDTY_12.1_* gave effect to increase of root branching and water uptake by root. Result of this study is in the same track previous researches where they also reported effect of this major-effect *qDTY* to improvement of traits related to drought tolerance [11,26,34].

Evaluation of selected PLs showed UKMG17 and UKMG18 produced average yield with YA more than 1000 kg ha^−1^. YA of 1000 kg ha^−1^ and more will give significant profit for farmers [2]. Higher PH of selected MRQ74 PLs compared to MRQ74 showed that tugor pressure of MRQ74 unable to be maintained and affected cell development [35,36]. UKMG12 and UKMG15, which have higher GY than MR219 and great performance under both conditions and will be further tested in several locations and suggested to be cultivated by farmers. Selected MRQ74 PLs, which had *qDTY_12.1_* also possessed better RL and RW thus, confirmed the effect of *qDTY_12.1_* either present as single or combined with other *qDTYs* to the improvement of root traits under NS and RS. Longer RL and higher root density is highly crucial for rice to increase water uptake, avoid dehydration and increase resistance towards water deficiency [37,38]. While early flowering under RS is advantageous [39] because early flowering genotype able to escape late occurring RS [40,41], all selected MRQ74 PLs have delayed DTF yet their GY still higher than earlier flowering MRQ74. This situation agreed to what Romyen et al. [42] stated that early flowering is often associated with GY reduction. Previous study found that MR219 was very sensitive to RS compared to MRQ74, because it did not flowered under severe stress [15]. Water deficiency prior to reproductive stage caused delay in flowering time [43]. Earlier DTF of MR219 PLs showed that they were less affected by RS and were superior compared to their recurrent parent, MR219. Early flowering trait is one of the mechanisms of tolerance to escape from the effect of prolonged drought which happened at the most critical stage of growth and phenological development [44]. In addition, Fukai et al. [40] and Pantuwan et al. [41] also mentioned that rice cultivars which flowered earlier have the tendency to escape drought which will happen at the end of season and the GY are better than the cultivars which flowered later under the same level of drought. Delay in flowering under RS is caused by the combination of slow flower development and reduction of panicle elongation rate [43]. Therefore, MR219 which had later DTF was more affected by RS which resulted in lower GY. High heritability values for DTF indicates that this trait is highly dependent on the genetic factor. The differences in flowering time of MRQ74 PLs and MR219 PLs under RS suggests that this trait is also genotype-dependent. Reduction of PH and NP under RS were expected as also reported by Muhammad Ashfaq et al. [22]. Previous studies reported reduction of CC under RS [17,18,24] but that reduction will might not cause massive reduction of GY.

Higher GY was obtained in late maturing lines which caused by several episodes of heavy rains during the RS imposition period that help these lines to recover from the impact of RS on GY. However, previous studies recorded negative correlation between GY and DTF under severe RS condition where the late flowering genotypes gave lower yield than early flowering genotypes [15,16,45,46]. Low positive correlation between PH and GY also recorded before [15,16,47], which indicates that the tall plants are capable to produce more GY compared to shorter plants. However, if the plant is too tall, it will be prone to lodging because of wind. MRQ74 and its PLs had a semi-dwarf to medium PH, which was desired. Increased RL and RW helped plant to gain more nutrients and increased its ability to obtain more water [48] for development and the most important process for plants, photosynthesis. Positive correlation between these two traits with GY is desired where breeders will select drought tolerant genotypes with high RL and RW for further trials. Traits that were greatly associated with drought should have positive correlation with GY in order for the plants to have better drought tolerance. Based on correlation between CC and GY, any increase or decrease in CC was not related to GY under RS due to the insignificant correlation value. Selection of drought tolerant genotypes based on CC is not favorable because of this condition. NP recorded a strong positive correlation with GY under NS and RS, which means that GY was dependent on how much tiller the genotypes can produce. RW and RL recorded high positive correlation in this study, which agreed to the result obtained by Venuprasad et al. [49]. 

Selection of GY, which is a complex trait under RS is less effective since heritability (*H*) is low [31,50]. However, high H of MRQ74 PLs and MR219 PLs for GY under RS recorded in this study indicated that this trait was affected by genetics and the probability for the genotypes to produce high yield if cultivated in different environments also will be high. Several experiments conducted at IRRI and elsewhere have clearly demonstrated that grain yield has moderate to high broad sense *H* under drought [2]. Furthermore, the *H* of GY was comparable with the *H* of secondary yield traits or physiological traits under RS [5] thus confirming the appropriateness of selecting PLs with high GY under RS for future studies. Apart of that, moderate to high *H* for DTF, PH and LR of MRQ74 PLs and MR219 PLs under RS made these traits as favorable criteria in selection for drought tolerant genotypes.

## 4. Materials and Methods

### 4.1. Plant Materials

BC_1_F_7_ drought tolerant PLs with the background of MR219 and MRQ74 were used in this study. Both populations were derived from crosses between the recipient parents (MR219 and MRQ74) and three *qDTY* donors viz. IR77298-14-1-2-10 (*qDTY_2.2_*), IR81896-B-B-195 (*qDTY_3.1_*) and IR84984-83-15-18-B (*qDTY_12.1_*). We used 36 PLs of MR219 and 39 PLs of MRQ74 that have different combination of *qDTYs*. MR219 and MRQ74 were included as susceptible checks while donor parents as tolerant checks. MRQ74 is an aromatic and high-quality rice variety generated from crossings Q34/KASTURI//KDML///Q34 [51] and previously reported to have moderate tolerance to drought [16]. MR219 is a high-yielding mega-variety of Malaysia generated from a crossing between MR137 and MR151 [52] and reported to be highly-susceptible to drought [15]. MRQ74 and MR219 takes 105-111 and 125 days to reach maturity [51,52].

### 4.2. RS and NS Preparation and Experimental Design

Research plots in Universiti Kebangsaan Malaysia and Perak State Department of Agriculture (Titi Serong) were used for this study. Imposition of RS was done prior to reproductive stage, which is 30 days after transplanting 21 days old seedlings. Each hill contained one seedling at distance of 25 cm × 25 cm between rows and hills. Stagnant water was not allowed in the RS plot until maturity. For NS, water level of 5 cm was maintained from day of transplanting until a week before harvesting. Alpha-lattice design with two replications was used in this study. RS level was monitored using peiziometer and gravimetric method. 

### 4.3. Collection and Analysis of Morphological and Agronomical Traits

Each PLs and checks were evaluated for morphological traits namely days to 50% flowering (DTF), number of panicles (NP), chlorophyll content (CC), grain length (GL), root length (RL), root weight (RW) and leaf rolling score (LR). Agronomical traits collected were plant height (PH) and 100-grain weight (GW). All genotypes were harvested and grain yield (GY) weighed converted to kilogram per hectare (kg ha^−1^). All traits were collected using protocols described in Standard Evaluation System of Rice [53]. For LR, the scoring is based on the method published before by O’Toole and Cruz [54]. The score given was from 0 to 5 in which score 0 means the leaves did not rolling while score 5 means the leaves rolled completely. LR score recorded four weeks after RS imposition at 12:00 p.m. Fertlizers (90-40-40 NPK kg ha^−1^), insecticides and fungicides were applied at the rate of suggested by Department of Agriculture Malaysia. 

### 4.4. Genotyping for qDTY Validation

Plant Genetics Research Laboratory in Biological Sciences Building, Faculty of Science and Technology, Universiti Kebangsaan Malaysia was the main place for all molecular works. Fresh leaves of each genotype were collected and ground in 2.0 µL microcentrifuge tubes submerged in liquid nitrogen using TissueLyser II machine (QIAGEN, Germantown, MD, USA). Genomic DNA extraction were carried using cetyltrimethyl ammonium bromide (CTAB) protocol as described by Murray and Thompson [55]. Quantity and quality of DNAs were analyzed using agarose gel electrophoresis and Nanodrop Spectrophotometer for more precise quantification. DNA was further diluted with 1× Tris-EDTA (TE) buffer to concentration of 25 ng/µL as EDTA which contained in the buffer inhibit DNAses and prevent degradation of DNA. 

Polymerase chain reaction (PCR) using 20 simple sequence repeat (SSR) primers (eight for *qDTY_2.2_*, three for *qDTY_3.1_* and nine for *qDTY_12.1_*) was done for all genotypes. Total PCR mixture of 15 µL comprises of 1.5 µL 10× Mg^2+^ free buffer, 0.9 µL of 25 mM MgCl_2_, 1.2 µL of 10 mM dNTPs, 0.6 µL each 10 mM forward and reverse primers, 9.0 µL double deionized water, 1 µL of 25 ng/µL DNA template and 0.2 µL *Taq* DNA polymerase (5 U/µL). PCR plates with 96 wells were used and sealed with sealing film to prevent evaporation of PCR mixture. Amplification reaction was carried out using Eppendorf Mastercycler Nexus Gradient machine (Eppendorf, Hamburg, Germany) and the following profile was applied: initial denaturation at 95 °C for 4 min, followed by 30 cycles of denaturation at 95 °C for 45 s, annealing at 55–60 °C depending on primers for 45 s and extension at 72 °C for 45 s; and final extension at 72 °C for 5 min. Polyacrylamide gel electrophoresis using 8% gel in 1× Tris-Borate-EDTA (TBE) buffer was done to analyze the amplicons at 100 V and running dependent on expected product sizes of SSR markers. 

### 4.5. Analysis of Variances, Heritability, Correlation and qDTY Class Analysis

Differences of all genotypes and heritability for traits collected in this study were carried out using PB Tools 1.4. *T*-test was used to differentiate each trait collected for each genotype in NS and RS conditions using Minitab 17. Correlation coefficients between traits were calculated and visualized using Corrplot package in R Studio. Mixed-model analysis using Minitab 17 was carried out to evaluate the effect of each *qDTY* and *qDTYs* combinations to morphological and agronomical traits recorded. The following model was used:

$$y_{ijkl}=\mu +r_{k}+b{\left(r\right)}_{kl}+q_{i}+g{\left(q\right)}_{ij}+e_{ijkl}$$

In which *µ* is the mean of the population, *r_k_* is the effect of kth replicate, *b*(*r*)*_kl_* is the effect of the *l*th block within the *k*th replicate, *q_i_* is the effect of *i*th *qDTY*, *g*(*q*)*_ij_* is the effect of the *j*th genotype nested within the *i*th *qDTY* and *e_ijkl_* is the error. Fixed effects are *qDTYs* and genotypes within *qDTY* while random effects are replicate and blocks within replicates.

## 5. Conclusions

We confirmed the presence of *qDTYs* in PLs improved morphological, agronomical traits and GY under NS and RS. *qDTY_12.1_* individually affected most number of traits of both populations which confirmed the consistentcy of this *qDTY*’s effect in different genetic backgrounds. However, *qDTY_12.1_* needs to interact with other *qDTY*/*s* to enhance GY of these two PL populations. Its presence under NS and RS does not need to be accompanied by other *qDTY* for the improvement of secondary traits such as RW and RL, but necessary if selection for high GY is to be made. Since this *qDTY*’s effect is consistent in these two Malaysian rice cultivars, other high yielding but drought susceptible cultivars can be introgressed with this *qDTY* to improve GY. Selected PLs with high GY and favourable phenotypes in this study can be tested in different environments to further confirm their performance and stability.

## Figures and Tables

**Table 1:** Mean values of traits of MRQ74 drought yield quantitative trait loci *(qDTY)* classes under NS and RS.

Traits	Environment	*qDTY* Classes							
		A	B	C	D	E	F	G	MRQ74
DTF	NS	87.17 c	91.00 ab	89.67 b	84.63 d	88.75 b	76.20 e	75.67 e	93.00 a
	RS	90.92 a	91.50 ab	86.33 abc	84.06 bc	91.00 a	79.70 c	80.00 c	89.00 abc
PH	NS	54.17 bc	48.00 c	63.33 a	54.00 bc	55.63 b	52.00 bc	54.67 bc	48.00 c
	RS	58.14 bc	48.32 de	65.75 a	55.49 cd	60.43 ab	47.57 c	55.95 bcd	58.17 abcd
NP	NS	10.63 ab	6.50 ab	8.50 ab	11.81 a	9.75 ab	7.10 b	7.33 ab	12.50 ab
	RS	10.75 a	6.50 a	8.17 a	11.19 a	11.63 a	8.80 a	10.00 a	14.50 a
CC	NS	43.65 c	43.55 bc	43.70 bc	44.08 bc	43.28 c	47.16 a	45.53 ab	42.20 c
	RS	42.23 c	41.27 bc	42.23 bc	42.98 bc	42.09 bc	44.51 a	43.92 ab	42.60 abc
LR	RS	1.79 bc	1.00 cd	1.17 d	1.94 b	1.50 bcd	2.00 b	2.00 b	3.00 a
RW	NS	2.05 c	1.66 f	1.66 f	1.95 d	2.27 b	1.67 f	1.86 e	2.81 a
	RS	2.24 b	1.53 bc	1.94 bc	2.20 b	2.31 b	1.50 c	1.83 bc	3.66 a
RL	NS	9.72 c	7.72 f	7.40 g	9.35 d	11.08 b	7.65 f	8.53 e	14.17 a
	RS	12.08 a	9.73 ab	10.95 ab	11.61 d	12.78 a	9.25 b	13.02 a	13.88 a
GW	NS	20.30 b	15.50 d	21.80 a	18.80 c	19.30 c	20.60 b	22.50 a	18.40 c
	RS	19.20 cd	16.90 d	24.10 a	19.60 c	20.10 bc	21.30 b	23.30 a	17.2 d
GL	NS	0.90 a	0.80 a	0.92 a	1.21 a	0.89 a	0.89 a	0.95 a	0.85 a
	RS	0.90 b	0.90 ab	0.96 a	0.89 b	0.92 ab	0.94 a	0.93 ab	0.89 ab
GY	NS	2715.36 c	1446.93 e	2316.93 d	3381.47 b	2460.30 d	1882.03 e	1711.60 e	4361.20 a
	RS	920.81 c	521.85 cd	1104.75 bc	976.90 bc	1826.58 a	445.85 d	915.88 bc	1618.06 ab

Mean values with the same letter at each row are not significantly different by LSD (*p* < 0.05), days to 50% flowering (DTF), plant height (PH) in cm, number of panicles (NP), chlorophyll content (CC), leaf rolling (LR), root weight (RW) in g, root length (RL) in cm, grain weight (GW) in g, grain length (GL) in cm, grain yield (GY) in kg ha^−1^.

**Table 2:** Mean values of traits of MR219 *qDTY* classes under NS and RS.

Traits	Environment	*qDTY* Classes						
		A	B	C	D	E	F	MR219
DTF	NS	91.33 d	85.90 e	94.00 c	83.80 f	86.00 e	95.50 b	107.00 a
	RS	92.50 bc	85.85 de	88.50 cd	84.20 e	86.00 de	95.63 ab	98.00 a
PH	NS	62.00 bc	60.30 c	50.75 e	57.00 d	69.00 a	55.00 de	68.00 ab
	RS	63.29 b	59.33 c	58.17 cd	55.08 d	64.00 bc	55.38 d	76.67 a
NP	NS	7.33 a	7.50 a	5.00 bc	5.77 b	8.33 a	4.50 c	5.67 abc
	RS	8.03 ab	8.17 a	9.42 a	6.47 b	6.67 ab	6.08 b	10.33 a
CC	NS	40.38 a	41.00 a	40.90 a	41.83 a	39.43 a	39.53 a	39.00 a
	RS	37.58 c	39.80 ab	39.42 abc	40.78 a	39.00 abc	36.30 c	36.17 bc
LR	RS	0.58 abc	0.39 bc	0.50 abc	0.68 a	0.67 abc	0.71 ab	0.00 c
RW	NS	0.58 b	0.76 ab	0.80 ab	0.68 ab	0.67 ab	0.66 ab	1.36 a
	RS	0.58 ab	0.38 bc	0.41 c	0.34 c	0.90 a	0.56 abc	0.82 ab
RL	NS	15.53 ab	14.52 abc	13.63 abc	13.38 c	12.55 bc	13.95 bc	18.00 a
	RS	13.41 ab	11.16 bc	12.63 abc	9.88 c	16.78 a	12.22 abc	13.38 abc
GW	NS	19.30 a	18.80 a	14.50 c	19.40 a	19.10 ab	17.40 b	16.70 bc
	RS	19.60 a	19.20 a	19.50 a	18.60 a	19.60 ab	20.00 a	13.50 b
GL	NS	0.93 a	0.97 a	0.98 a	0.95 a	0.97 a	0.95 a	1.02 a
	RS	0.92 a	0.96 a	0.97 a	0.94 a	0.93 a	0.92 a	0.97 a
GY	NS	2702.31 a	2269.58 b	1811.84 bc	2269.58 b	2364.53 abc	1708.23 c	2286.23 abc
	RS	2753.22 a	1992.53 b	1353.31 c	1438.75 c	2788.62 a	1323.47 c	2005.71 b

Mean values with the same letter at each row are not significantly different by LSD (*p* < 0.05), days to 50% flowering (DTF), plant height (PH) in cm, number of panicles (NP), chlorophyll content (CC), leaf rolling (LR), root weight (RW) in g, root length (RL) in cm, grain weight (GW) in g, grain length (GL) in cm, grain yield (GY) in kg ha^−1^.

**Table 3:** Heritability of each trait in each population under NS and RS.

Traits	MRQ74 PLs		MR219 PLs	
Environment	NS	RS	NS	RS
DTF	0.99	0.66	0.99	0.83
PH	0.89	0.86	0.80	0.88
NP	0.68	0.34	0.88	0.48
CC	0.69	0.55	0.67	0.53
LR	NA	0.63	NA	0.49
RW	0.99	0.14	0.00	0.10
RL	1.00	0.33	0.69	0.39
GW	0.93	0.85	0.92	0.00
GL	0.05	0.53	0.99	0.99
GY	0.98	0.87	0.91	0.96

Days to 50% flowering (DTF), plant height (PH), number of panicles (NP), chlorophyll content (CC), leaf rolling (LR), root weight (RW), root length (RL), grain weight (GW), grain length (GL), grain yield (GY), non-stress (NS), drought stress (RS), not available (NA).

## Supplementary Materials

The following are available online at https://www.mdpi.com/2223-7747/8/6/186/s1, Table S1: Morphological and agronomical traits of Selected MRQ74 PLs under RS, Table S2: Morphological and agronomical traits of Selected MRQ74 PLs under NS, Table S3: Morphological and agronomical traits of Selected MR219 PLs under RS, Table S4: Morphological and agronomical traits of Selected MR219 PLs under NS, Table S5: Correlation between traits in MRQ74 PLs, Table S6: Correlation between traits in MR219 PLs.
