# Supplementary material for: Drought Yield QTL (qDTY) with Consistent Effects on Morphological and Agronomical Traits of Two Populations of New Rice (Oryza sativa) Lines
Source: Plants (Basel). 2019 Jun 24;8(6):186. doi: 10.3390/plants8060186 (PMC6630983; doi:10.3390/plants8060186)
Supplement: Supplementary file 1 [file plants-08-00186-s001.pdf]

Supplementary Table 1**.** Morpho-physiological and agronomical traits of Selected MRQ74 PLs under RS

Genotypes	*qDTY/s*	RW	RL	LR	GW	GL
UKM-G16	$qDTY_{12.1}$+$qDTY_{3.1}$	2.53a	14.03a	1.50b	20.90b	0.97a
UKM-G18	$qDTY_{2.2}$	2.10a	12.05a	1.50b	18.70c	0.82b
UKM-G17	$qDTY_{12.1}$+$qDTY_{2.2}$	2.91a	16.38a	2.00ab	21.60b	0.89ab
MRQ74	No *qDTY*	3.66a	13.88a	3.00a	17.20d	0.89ab

Same letters in each column indicate non-significant different (LSD, p<0.05), leaf rolling (LR), root weight (RW) in g, root length (RL) in cm, grain weight (GW) in g, grain length (GL) in cm.

Supplementary Table 2. Morpho-physiological and agronomical traits of Selected MRQ74 PLs under NS

Genotypes	*qDTY/s*	RL	RW	GW	GL
UKM-G16	$qDTY_{12.1}$+$qDTY_{3.1}$	11.33b	2.32a	19.40bc	1.05a
UKM-G18	$qDTY_{2.2}$	6.40c	1.46b	19.10c	3.45a
UKM-G17	$qDTY_{12.1}$+$qDTY_{2.2}$	13.58e	2.72e	21.00ab	0.85a
MRQ74	No *qDTY*	14.17a	2.81f	18.40c	0.85a

Same letters in each column indicate non-significant different (LSD, p<0.05), root weight (RW) in g, root length (RL) in cm, grain weight (GW) in g, grain length (GL) in cm.

Supplementary Table 3. Morpho-physiological and agronomical traits of Selected MR219 PLs under RS

Genotypes	*qDTY/s*	RL	RW	GW
UKM-G12	$qDTY_{12.1}$+$qDTY_{3.1}$+$qDTY_{2.2}$	12.23a	0.69a	1.71bc
UKM-G13	$qDTY_{12.1}$+$qDTY_{3.1}$+$qDTY_{2.2}$	13.08a	0.74a	2.19a
UKM-G15	$qDTY_{3.1}$+$qDTY_{2.2}$	10.75a	0.24a	2.17a
UKM-G14	$qDTY_{12.1}$+$qDTY_{3.1}$	10.75a	0.16a	1.88ab
MR219	No *qDTY*	13.38a	0.82a	1.35c

Same letters in each column indicate non-significant different (LSD, p<0.05), root weight (RW) in g, root length (RL) in cm, grain weight (GW) in g.

Supplementary Table 4. Morpho-physiological and agronomical traits of Selected MR219 PLs under NS

Genotypes	*qDTY/s*	RL	RW	GW
UKM-G12	$qDTY_{12.1}$+$qDTY_{3.1}$+$qDTY_{2.2}$	10.00d	0.68a	1.67b
UKM-G13	$qDTY_{12.1}$+$qDTY_{3.1}$+$qDTY_{2.2}$	13.15cd	0.46a	2.07a
UKM-G15	$qDTY_{3.1}$+$qDTY_{2.2}$	14.55bc	0.70a	2.28a
UKM-G14	$qDTY_{12.1}$+$qDTY_{3.1}$	15.75bc	0.57a	1.66b
MR219	No *qDTY*	18.00ab	1.58a	1.59b

Same letters in each column indicate non-significant different (LSD, p<0.05), root weight (RW) in g, root length (RL) in cm, grain weight (GW) in g.

Supplementary Table 5. Correlation between traits in MRQ74 PLs

Traits	DTF		PH		NP		CC		RW		RL		LR	GW		GL	
Ecosystem	NS	RS	NS	RS	NS	RS	NS	RS	NS	RS	NS	RS	RS	NS	RS	NS	RS
PH	0.51*	0.40*															
NP	0.25*	0.37*	0.38*	0.38*													
CC	-0.61*	-0.29*	-0.22*	-0.21	-0.15	-0.02											
RW	0.21	0.16	0.01	0.27*	0.00	0.27*	-0.41*	-0.18									
RL	0.21	0.16	0.01	0.21	0.00	0.14	-0.42*	-0.18	0.99	0.68							
LR	NA	-0.03	NA	-0.07	NA	0.02	NA	0.07	NA	-0.11	NA	-0.15					
GW	-0.17	-0.29*	0.14	0.09	0.04	-0.34*	0.32*	0.16	-0.48*	-0.23	-0.53*	-0.15	-0.21				
GL	0.14	-0.03	0.07	0.03	0.12	-0.09	-0.02	0.23	-0.12	-0.03	-0.12	-0.09	-0.17	-0.01	0.27*		
GY	0.46*	0.46*	0.35*	0.61*	0.46*	0.68*	-0.22	-0.23	0.22	0.26*	0.23	0.15	-0.10	-0.06	-0.12	0.05	-0.13

*Significant at $p<0.05$, days to flowering (DTF), plant height (PH) in cm, number of panicles (NP), chlorophyll content (CC), leaf rolling (LR), root weight (RW) in g, root length (RL) in cm, grain weight (GW) in g, grain length (GL) in cm, grain yield (GY) in $kgha^{-1}$.

Supplementary Table 6. Correlation between traits in MR219 PLs

Traits	DTF		PH		NP		CC		RW		RL		LR	GW	
Ecosystem	NS	RS	NS	RS	NS	RS	NS	RS	NS	RS	NS	RS	RS	NS	RS
PH	0.02	0.09													
NP	-0.10	-0.15	0.33*	0.62*											
CC	-0.33*	-0.63*	0.36*	-0.18	-0.03	0.10									
RW	0.01	0.11	0.01	0.46*	-0.10	0.52*	0.04	-0.18							
RL	0.31*	0.23	0.01	0.40*	0.02	0.24	0.05	-0.34*	0.06	0.23					
LR	NA	-0.25	NA	0.22	NA	0.31*	NA	-0.06	NA	0.23	NA	0.09			
GW	-0.27*	0.07	0.32*	0.34*	0.26*	0.20	0.39*	-0.15	-0.11	0.08	0.00	0.15	0.28*		
GY	0.20	0.25	0.45*	0.51*	0.42*	0.35*	0.20	-0.43*	-0.10	0.33*	0.06	0.37*	0.03	0.34*	0.25*
GL	-0.07	0.04	0.00	-0.03	0.07	-0.12	0.00	0.00	0.29*	-0.11	0.04	-0.12	0.11	-0.12	0.06

*Significant at $p<0.05$, days to flowering (DTF), plant height (PH) in cm, number of panicles (NP), chlorophyll content (CC), leaf rolling (LR), root weight (RW) in g, root length (RL) in cm, grain weight (GW) in g, grain yield (GY) in $kgha^{-1}$.
